# The Wheat Grain Contains Pectic Domains Exhibiting Specific Spatial and Development-Associated Distribution

**DOI:** 10.1371/journal.pone.0089620

**Published:** 2014-02-21

**Authors:** Anne-Laure Chateigner-Boutin, Brigitte Bouchet, Camille Alvarado, Bénédicte Bakan, Fabienne Guillon

**Affiliations:** Unité de Recherche 1268 Biopolymères Interactions Assemblages, INRA, Nantes, France; Iowa State University, United States of America

## Abstract

Cell walls are complex structures surrounding plant cells with a composition that varies among species and even within a species between organs, cell types and development stages. For years, cell walls in wheat grains were described as simple walls consisting mostly of arabinoxylans and mixed-linked beta glucans. Proteomic and transcriptomic studies identified enzyme families involved in the synthesis of many more cell wall polysaccharides in the wheat grains. Here we describe the discovery of pectic domains in wheat grain using monoclonal antibodies and enzymatic treatment to degrade the major cell wall polymers. Distinct spatial distributions were observed for rhamnogalacturonan I present in the endosperm and mostly in the aleurone layer and homogalacturonan especially found in the outer layers, and tight developmental regulations were unveiled. We also uncovered a massive deposition of homogalacturonan via large vesicular bodies in the seed coat (testa) beneath a thick cuticle during development. Our findings raise questions about the function of pectin in wheat grain.

## Introduction

The wheat grain is a major source of food and animal feed rich in polysaccharides. These polysaccharides are beneficial for human health as dietary fibre and are important for the processing quality of the grain including milling and bread-making quality [1]. On the contrary, detrimental effects are associated with wheat grain polysaccharides for (monogastric) animal production [2]. For the plant, the endosperm is a storage tissue within the wheat grain where carbohydrates accumulate during development to provide energy and nutrients to the seedling upon germination. The main component of wheat endosperm is starch (70–80%). Cell wall polysaccharides account for only about 3% of the starchy endosperm but have major impacts on its end-use properties. Arabinoxylans and mixed-linked glucans ((1–3)(1–4)-β-D-glucans) are the main cell wall polysaccharides of the wheat starchy endosperm (70% and 20% of cell wall polysaccharides respectively), while the remaining percents consist of cellulose and mannans [3]–[6].

Recent studies revealed that in developing wheat and barley, callose and xyloglucans are deposited transiently in the cell wall of the endosperm [7]–[8]. These discoveries were made possible by the availability of antibodies specific to domains of cell wall polysaccharides. These studies also revealed that upon development the detection profiles of several polysaccharides varied; some polysaccharides (mannans) were detected specifically in the starchy endosperm while others such as xyloglucans were detected only in the cells of the modified aleurone layer in the crease region also called transfer cells.

Strikingly, no pectin was ever reported in wheat grain and early works on the composition of wheat flour failed to detect pectin [3], [5]. Conversely, pectin has been reported in the endosperm of rice and of *Brachypodium distachyon*, which are closely related to wheat [9]–[10].

Because of the apparent simplicity of the cell wall contained within the starchy endosperm, we proposed the wheat grain as a model to identify the cellular machinery responsible for the biosynthesis of the cell wall. The biosynthesis of cell wall polysaccharides requires many enzyme activities that are carried out mostly by proteins of the glycosyltransferase (GT) superfamily. This superfamily is divided into 95 GT families based on sequence similarity and listed in the Carbohydrate Active Enzyme (CAZY) database [11]. In the recent years, many enzyme activities have been associated to GT families, some as a result of the analysis of organs particularly enriched in one glycan (e.g; nasturtium seed, cotton flower) and others via mutant analysis mostly in *Arabidopsis thaliana*, but also in grasses [12]–[13]. A few activities have been monitored on heterologously expressed proteins [12]–[13]. The activity of members of several GT families has been guessed based on assertions about the family and clade within the family they belong to [7], [13].

In a transcriptomic survey of the developing wheat endosperm, Pellny et al [7] detected transcripts belonging to GT families implicated in the synthesis of xylans, mixed-linked glucans, cellulose, mannans, xyloglucans and callose. Surprisingly, they also identified several GT families involved in the synthesis of pectins (GT8, GT47, GT77). Likewise, we conducted a proteomic analysis of wheat endosperm fractions obtained at a stage of active cell wall deposition and enriched in Golgi apparatus, which is the site of synthesis of pectin and hemicelluloses. We were able to detect 64 putative GTs and among them several potentially involved in pectin synthesis [14]. We therefore decided to re-investigate the possibility that the wheat grain endosperm may contain pectin.

Pectin is a very complex polysaccharide composed of different domains covalently linked one to another [15]. Homogalaturonan (HG) is a homopolymer of α-1,4-linked galacturonic acid residues, xylogalacturonan (XGA) consists in a backbone of galacturonic acid substituted with xylose, rhamnogalacturonan I (RGI), is made of a backbone of repeating disaccharide units of rhamnose and galacturonic acid with side chains of arabinans, galactans and arabinogalactans and rhamnogalacturonan II (RGII) consists in a backbone of galacturonic acid with highly complex side chains. Furthermore some pectic domains can be methylated, acetylated and/or feruloylated by the action of transferases. These modifications impact the biological functions and technological properties of pectin since they affect the capacity of pectic chains to bind one to another [16]–[19].

Using immunolabeling targeting several pectic domains we unambiguously detected pectin in the endosperm and also in the outer layers of the wheat grain, and we highlighted tight spatial and developmental regulations. In addition, we observed impressive intracellular bodies in the seed testa filled with HG and pectin deposition in a thick layer underneath the cuticle.

## Materials and Methods

### Plant materials

*Triticum aestivum* cv. Recital was grown in pots in a greenhouse under conditions of natural day length (UMR Amélioration des Plantes et Biotechnologies Végétales, INRA-Rennes, France). The wheat seedlings were vernalized for 2 months in a growth chamber at 8°C then transplanted into individual pots containing a standard potting mixture (peat RHP15 Klassman, K Klassman France, Bourgoin Jallieu, France). An Osmocote (R) Exact Tablet containing Nitrogen (15%), Phosphate (9%), Potassium hydroxide (9%) and Magnesium (3%) (Scotts International B. V., Waardenburg, The Netherlands) was added. The plants were watered daily. To harvest grains at defined developmental stages, individual ears were tagged at flowering. Seed development was calculated on the basis of cumulated temperature in Celsius degrees days (°D) after flowering. The mean daily temperatures were recorded and the thermal times which corresponded to the temperature accumulated daily from anthesis, were calculated [20].

Grains were harvested at different stages of development: 45°D (cell division: 2 days post anthesis or DPA), 90°D (cell division: 5 DPA), 150°D (cell division: around 8 DPA), 250°D (differentiation, accumulation of storage products: around 11 DPA), 350°D (accumulation of storage products: around 17 DPA), 450°D (slow accumulation of storage product: around 23 DPA) and 750°D (mature grain beginning of desiccation: around 40 DPA). Only the grains from the middle of the ears were harvested. The embryo and the crease regions were not investigated in the course of this study.

### Monoclonal antibodies

One mouse monoclonal antibody: INRA RU1 [21], and four rats monoclonal antibodies LM6 [22], LM5 [23], LM19 and LM20 [24] were used. The antibody INRA RU1 binds specifically to the RGI backbone. The LM6 antibody recognizes (1–5)-α-L-arabinan, and LM5 recognizes a linear tetrasaccharide made of (1–4)-β-D-galactan. LM19 has a preference for and binds strongly to un-esterified HG. LM20 recognizes methyl-esters of HG and does not bind to un-esterified HG.

### Microscopy

#### Sample preparation

Pieces of tissue (1 mm^3^) were sampled from half wheat grains then prepared for analysis by transmission electron microscopy by fixing in 1% (v/v) glutaraldehyde and 3% formaldehyde in 0.1 M phosphate buffer, pH 7.4 overnight at 4°C. After fixation, tissues were rinsed 5 times with 0.1 M phosphate buffer, then deionized water, followed by dehydration through a graded aqueous ethanol series (30, 50, 70, 85, 95 and 100%). The samples were then progressively infiltrated with LRW (London Resin White acrylic) according to the following schedule: 20, 40, 60 and 80% LRW/ethanol and overnight in pure LRW. The infiltrated samples were finally embedded by LRW resin and polymerized for 48 h at 60°C.

#### Toluidine Blue and Sudan red staining

Semi-thin sections (1 µm, ultracut UC7, LEICA) were cut from grain embedded in LRW resin. Sections were stained with toluidine blue O 0.1% w/v for 10 sec or with Sudan red 1% in P20 polyethylene glycol to reveal the cuticles for 10 min, then washed and mounted on glass slides. Sections were examined with a Zeiss (Axiovert 135) microscope.

#### Differential interference contrast (DIC) microscopy

Cross-sections of wheat grains at the different stages of development were observed using differential interference contrast microscopy (Leica DMRD) to verify the quality of the sections (Fig. S6) and to identify the cell layers. The microscope was equipped with standard DIC optics using a Plan-APO 20X objective. It was coupled to a Nikon DS-1QM camera.

#### Immunofluorescence microscopy

Semi-thin sections (1 µm,) were cut from LRW embedded samples and collected on glass. To increase accessibility to pectic epitopes the sections were treated with lichenase (endo-1,3(4)-β-Glucanase) 40 U/ml (Megazyme International Ireland Ltd) overnight at 40°C and then with endo-1,4-β-Xylanase M6 (Megazyme International Ireland Ltd ) 50 U/ml overnight at 40°C in water. A set of immunolabeling was performed in parallel without enzymatic treatment for three stages (250°D, 350°D and 750°D) (Fig. S5) and with only xylanase or only lichenase for the stage 750°D. Pectin lyase 436 nkat/ml [25] in 50 mM acetate buffer pH 5 was applied overnight at 38°C on 750°D grain sections treated or not with xylanase and lichenase. Other 750°D grain sections were treated with xylanase and lichenase and then with 0.1 M sodium carbonate (Na_2_CO_3_) for 1 h at room temperature. Sections were incubated in a blocking solution of 3% (w/v) Bovine Serum Albumin (BSA) in 0.1 M phosphate-buffered saline (PBS, pH 7.2), to block non-specific labeling for 30 min at room temperature. Sections were then incubated with primary antibodies (LM5, LM6, LM19 and LM20 diluted at 1∶10, INRA RU1 diluted at 1∶3) in a PBS-Tween buffer (0.1 M PBS supplemented with 1% BSA and Tween 20 0.05%), for 1 h at room temperature. The sections were washed extensively in the PBS-Tween buffer and then incubated 1 h at room temperature in darkness with secondary antibodies (LM5, LM6, LM19, LM20: goat-anti-rat IgG; INRA RU1: goat-anti-mouse IgG) conjugated with Alexa Fluor 546 diluted 1∶100 (v/v) in the PBS-Tween buffer. Then, they were washed four times with PBS buffer, twice with deionized water and examined with a Leica (DMRD) microscope. A band-pass filter 515–560 nm was used as excitation filters and fluorescence was detected at >590 nm. For a given antibody, the same time of exposure was used.

Control experiments were performed omitting the primary antibodies on three stages (250°D, 350°D and 750°D) to check the absence of autofluorescence and demonstrate that the signal was due to the primary antibodies.

#### Immunolabeling in transmission electron microscopy (TEM)

Ultra-thin sections (80 nm, ultracut UC7, LEICA) were cut from LRW embedded samples and collected on nickel grids. Sections were blocked as described for immunofluorescence and then incubated in a solution containing the antibodies LM19 or LM20 diluted 1∶10 in the PBS-Tween buffer for 1 h at room temperature. The sections were extensively washed in PBS-Tween buffer and then incubated 1 h at room temperature in the dark with goat-anti rat antibody conjugated with 1 nm colloidal gold complexes diluted 1∶20 (v/v) in the PBS-Tween buffer. Labeling was then intensified with the silver enhancement kit (Aurion) according to the manufacturer's instructions then rinsed in deionized water. The ultra-thin sections were examined with a JEOL JEM 1230 transmission electron microscope with an accelerating voltage of 80 kV.

#### Periodic acid-thiosemicarbazide-silver proteinate staining (PATAg)

he PATAg treatment was performed as described by [26]. Ultra-thin sections were treated with 1% aqueous periodic acid (Merck; Denmark) for 30 min. Then, they were rinsed 5 times in deionized water before incubation in thiosemicarbazide (0.2%) (Merck; Denmark) diluted in 20% acetic acid for 17 h. Sections were rinsed 5 times in decreasing acetic acid concentrations (20, 10, 5 et 2.5%), 4 times in deionized water and stained with 1% aqueous silver proteinate (Prolabo; France) in the dark for 30 min. After rinsing 5 times in deionized water, sections were mounted on copper grids prior TEM observation. The ultra-thin sections were examined with a JEOL JEM 1230 transmission electron microscope with an accelerating voltage of 80 kV.

Immunolabelings were performed on at least two grains obtained from different plants.

## Results

### Immunolabeling revealed the presence of specific pectic domains within the mature wheat grain

**Figure 1:**
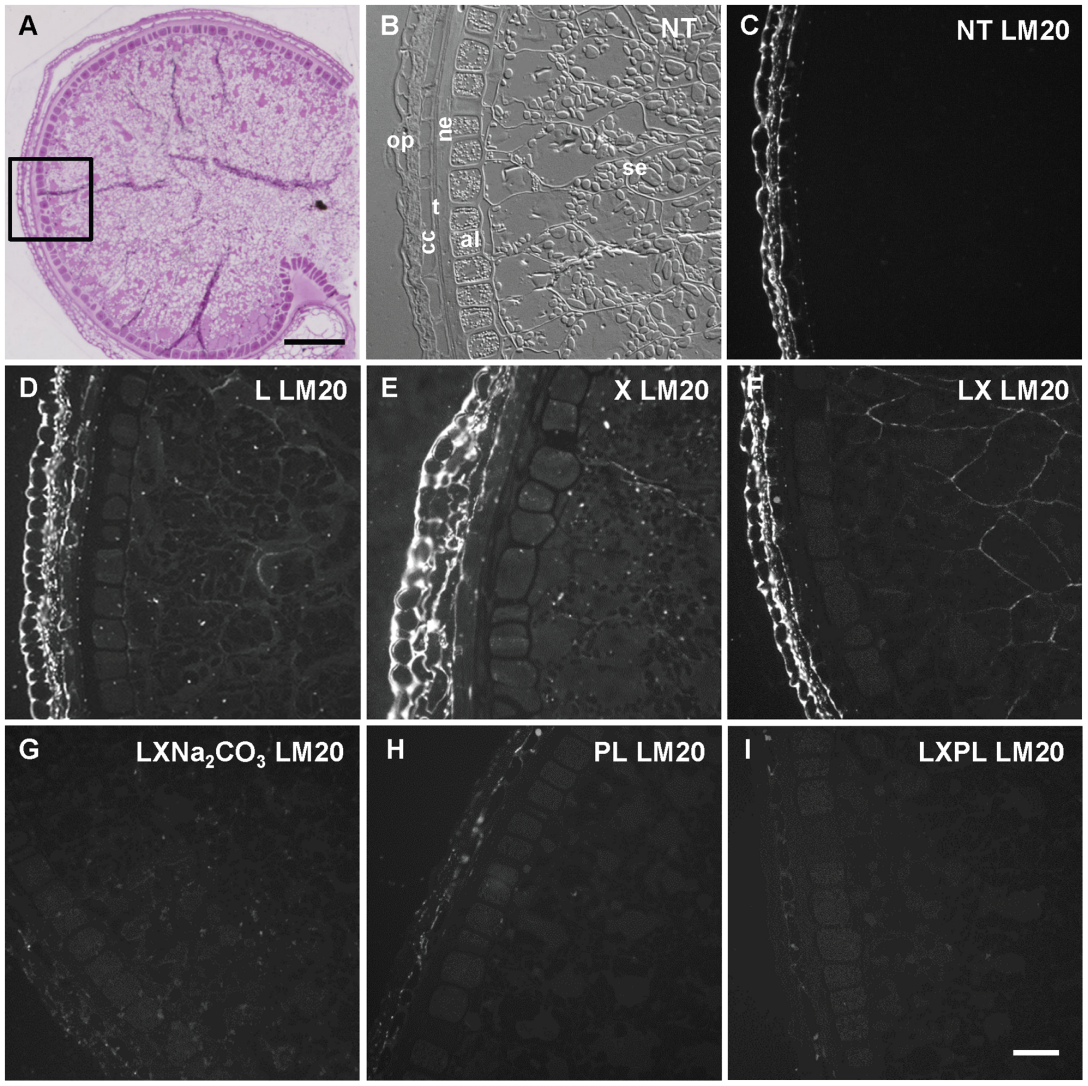
Homogalacturonan epitopes in wheat mature grain: effect of different treatments on LM20 labeling. A. Half wheat grain harvested at 750°D and stained with toluidine blue showing the tissues of the grain and the region where the immunofluorescence acquisitions were taken (black square). B. Differential interference contrast (DIC) showing the tissue structures. C, D, E, F, G, H, I. Immunofluorescence images localizing pectic LM20 epitope. C. The sections non treated (NT) to degrade arabinoxylans and beta-glucans exhibit labeling in the outer layers but no labeling in the endosperm. D, E. Similar results are obtained when the sections are treated only with lichenase (L) or only with xylanase (X). F,G. Incubation with lichenase and xylanase (LX) revealed LM20 labeling in the endosperm and outer layers (F) and removal of HG methylesters by Na_2_CO_3_ decreased LM20 labeling (G). H, I. Pectin lyase treatment which degrades HG decreased LM20 signal. al: aleurone layer, cc: cross cells, ne: nucellar epidermis, op: outer pericarp, se: starchy endosperm, t: testa,. Bars represent 250 µm for A, 50 µm for I (the same scale was applied for B, C, D, E, F, G and H).

The mature wheat grain (or caryopsis) undergoing desiccation within the spike consists mainly of the endosperm (starchy endosperm and aleurone layer) while the outer tissues are thin and compressed (Fig. 1A). The tissues surrounding the endosperm are of maternal origin. On the outside of the aleurone layer, lay the nucellar epidermis, which is the remnant of the nucellus, then the testa or seed coat, and the pericarp which can be divided into tube cells (rarely seen in cross sections), cross cells and outer pericarp (Fig. 1B).

Immunolabelings were performed on mature grain sections first with the antibody LM20 which recognizes HG. A fluorescent signal was obtained only in the outer layers of the grain (Fig. 1C). Since reports of epitope masking were published recently for cell wall epitopes [27], [28], the grain sections were treated with enzymes which degrade the endosperm abundant cell wall polymers arabinoxylans and mixed-linked glucans. When the grain sections were incubated with both enzymes, LM20 epitopes were detected in the grain outer layers as well as in the starchy endosperm cell walls (Fig. 1F) whereas when only one enzyme treatment was applied (lichenase only or xylanase only), no labeling was observed in the endosperm (Fig. 1D and Fig. 1E). Incubation with pectin lyase, an enzyme which degrades HG, drastically decreased LM20 labeling (Fig. 1H), and even more when the sections were treated with lichenase and xylanase (Fig. 1I). We noticed a change in the cell structure of the testa which appeared more crushed after lichenase, xylanase, and pectin lyase treatments (Fig. S1).

Lichenase treatment resulted in the complete loss of mixed-linked glucan labeling, and xylanase treatment decreased arabinoxylan labeling (Fig. S2). These results indicate that cell walls in mature wheat outer layers and endosperm contain HG and that in the endosperm HG epitopes react with HG antibodies when the abundant arabinoxylans and mixed-linked glucans are degraded. Variation in epitope accessibility between cell walls in outer layers and endosperm is probably due to the difference in composition and structure of the corresponding cell walls [6].

In the light of these results all the immunofluorescence experiments were subsequently performed on sections treated with both xylanase and lichenase.

LM20 recognizes HG preferentially with a high degree of methylesterification [24]. When grain sections were incubated with lichenase, xylanase and then with sodium carbonate, which removes methylesters, prior to immunolabeling with LM20 no signal was detected (Fig. 1G) indicating that HG in mature wheat grain is methylesterified.

**Figure 2:**
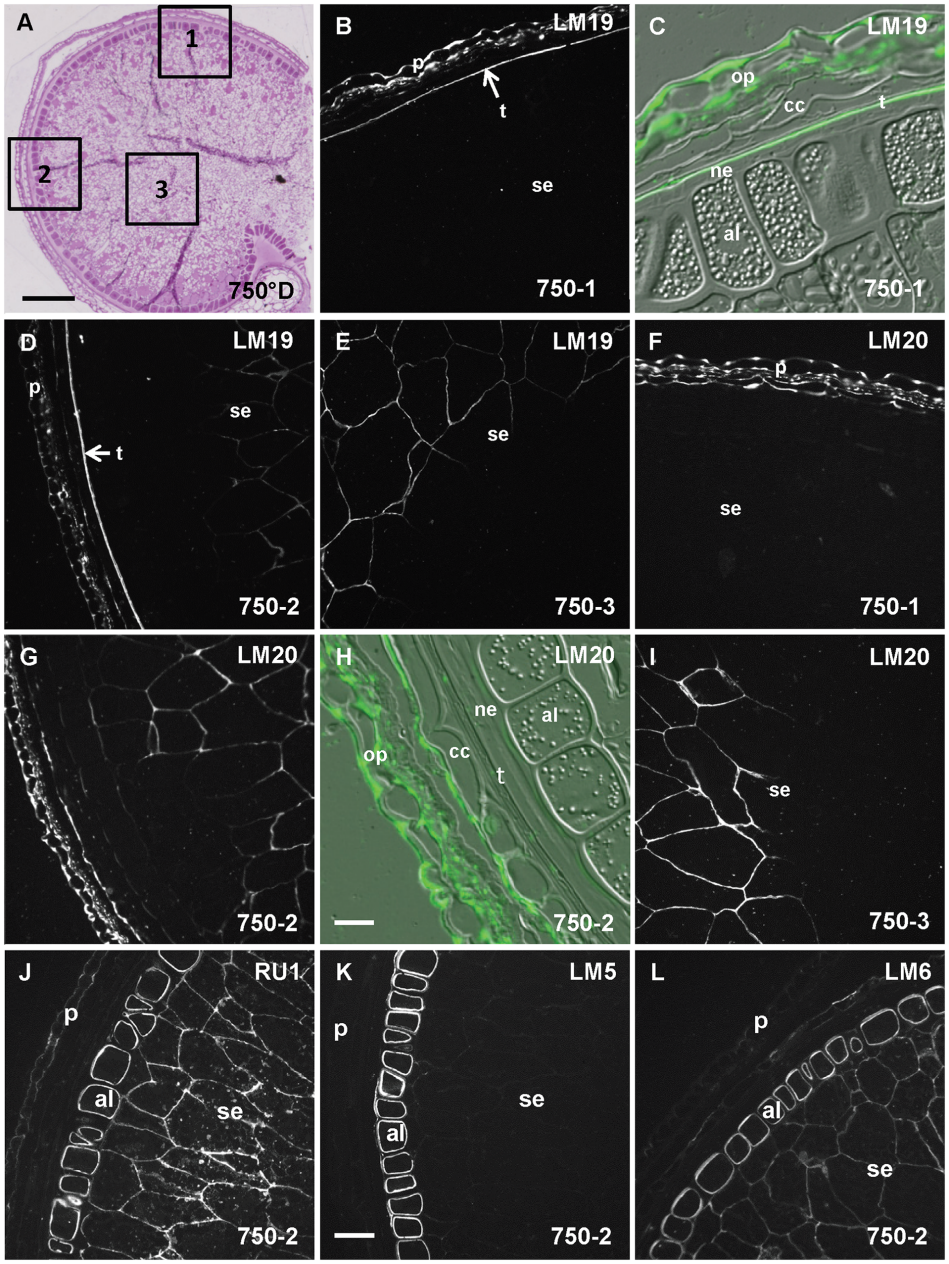
Pectic epitopes in wheat mature grain are heterogeneously distributed. A. Half wheat grain harvested at 750°D and stained with toluidine blue showing the tissues of the grain and the regions 1, 2 and 3 where the immunofluorescence acquisitions were taken. B, C, D, E, F, G, H, I, J, K L. Immunofluorescence images localizing low (or non) methylesterified HG epitope using LM19 antibody (B, C, D and E), methylesterified HG epitope using LM20 antibody (F, G, H, I), rhamnogalacturonan I backbone epitope (J) using RUI antibody or galactan and arabinan side chains epitopes using LM5 and LM6 antibodies (K and L). In C and H, immunofluorescence and DIC images are merged to identify the labeled layers, the testa (t) and outer pericarp (op) for LM19 in C, and the outer wall of the cross cells (cc) and outer pericarp for LM20 in H. The sections were all treated with lichenase and xylanase prior to immunolabeling. An heterogeneity of labeling is noticed in the starchy endosperm (se) for both antibodies, in the dorsal region 1 the starchy endosperm is not labeled and in the peripheral region 2 and central region 3, it is heterogeneously labeled. al: aleurone layer, ne: nucellar epidermis. Bars represent 250 µm for A, 50 µm for K (the same scale was applied for B, D, E, F, G, I, J and L), and 20 µm for H and C.

The starchy endosperm was heterogeneously labeled with LM20. Indeed, the cell walls were partially labeled in the peripheral (2) (Fig. 2G) and central (3) (Fig. 2I) starchy endosperm of the grain lobes but no signal was detected in the starchy endosperm of the dorsal (1) region (Fig. 2F). LM20 epitopes were not detectable in the aleurone layer. The grain outer layers were labeled (Fig. 2F, G and H). The signal was particularly strong in the walls of the outer pericarp and in the outer wall of the cross cells showing a polarity for these cells (Fig. 2H).

The antibody LM19 binds strongly and preferentially to un-esterified HG [24]. LM19 labeled the same regions than LM20 in the endosperm and the outer layers of the mature grain (Fig. 2B, D and E). In addition, a strong signal was obtained for the testa (Fig. 2B, C and D) with LM19. Part of the grain HG would be low or un-methylesterified. As expected, sodium carbonate treatment increased LM19 labeling (Fig. S3).

The antibody INRA RU1 binds specifically to the RGI backbone [21]. In the mature grain, INRA RU1 labeled the endosperm the signal was particularly strong in the aleurone layer (Fig. 2J). Zooming in the aleurone layer, the signal appeared more intense on the inside of the cell wall. The outer pericarp appeared labeled. RGI contains several types of side chains: arabinogalactan, arabinan and galactan side chains. We used antibodies LM6 and LM5 which bind to side chains of the RGI. LM6 binds to (1–5)-α-L-arabinan [22]; it labeled the endosperm with an intense signal in the aleurone layer and in contrast only a faint signal was obtained for the outer layers (Fig. 2L). The antibody LM5 was used to detect (1–4)-β-D-galactan [23] and revealed a signal only in the aleurone layer (Fig. 2K).

The positive immunolabelings obtained with all these pectin probes constitute evidence of the presence of pectic HG and RGI in the wheat mature grain undergoing desiccation. In addition, pectic HG signal was suppressed by a pectin lyase treatment. To further confirm the presence of pectin in mature grain we analyzed the monosaccharide composition of wheat grain. We detected traces of rhamnose and galacturonic acid (respectively 0.28% and 0.17% of whole grain alcohol insoluble fraction) which indicates the presence of pectin (Fig. S4).

Immunolabelings also highlighted a specific spatial distribution of the different pectic domains within the wheat mature grain with a predominant HG labeling in the outer layers, and a predominant RGI labeling in aleurone cells.

### Pectin deposition during grain development

**Figure 3:**
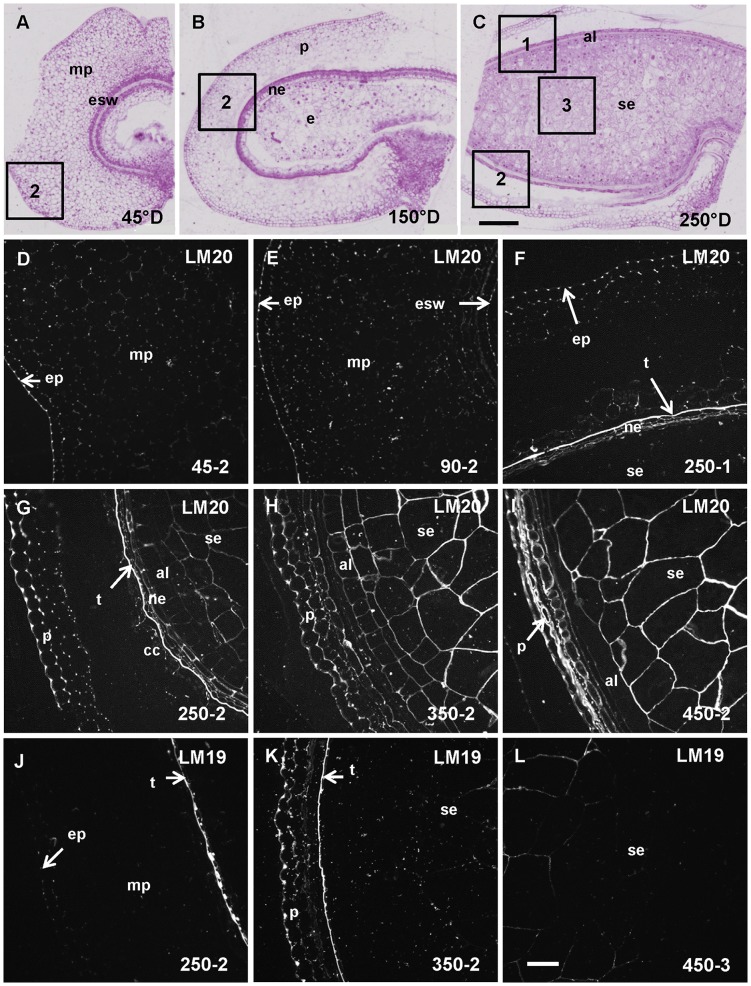
Homogalacturonan (LM20 and LM19 epitopes) deposition in developing wheat grain. A, B, and C. Half wheat grain harvested at 45°D, 150°D and 250°D stained with toluidine blue showing the evolution of the grain tissues and the regions 1, 2 and 3 where the immunofluorescence acquisitions were taken. D, E, F, G, H, I, J, K and L. Immunofluorescence images localizing methylesterified HG epitope using LM20 and low (or non) methylesterified HG epitope using LM19 antibody. The sections were all treated with lichenase and xylanase prior to immunolabeling. D. LM20 labeling is observed from 45°D in the pericarp and especially in the epiderm of the pericarp (ep). F, G. LM20 labeling is observed only at 250°D in the testa (t), and from 250°D in the endosperm (G, H, I). J. LM19 labeling is observed from 250°D especially in the testa, the pericarp is labeled from 250°D and the starchy endosperm (se) from 350°D (K). al: aleurone layer, cc: cross cells, e: endosperm, esw: embryo sac wall, mp: mesocarp, ne: nucellar epidermis, op: outer pericarp, p: pericarp, se: starchy endosperm. Bars represent 250 µm for A, B and C and 50 µm for the remaining images (all at the same scale).

At 45°D the grain is growing and consists mainly of the expanding pericarp (Fig. 3A). LM20 antibodies labeled the wheat grain at 45°D and an uneven labeling was observed (Fig. 3D). The outer grain layer of the pericarp was labeled continuously with brighter spots in the cell junctions. Parenchyma cell walls in the mesocarp (inner pericarp) were also labeled with a stronger signal in cell corners. Preferential labeling in the cell junctions was still observed at 250°D in the pericarp (Fig. 3F and G); at this stage the mesocarp degenerates as shown in Fig. 3C. In the outer layers at 250°D cell walls between two cross cells and between two nucellar epidermis cells were also labeled (Fig. 3G) and a particularly bright layer was observed corresponding to the testa. This bright signal was not observed for the other developmental stages. The developing endosperm was labeled with LM20 from 250°D (Fig. 3G), the labeling intensity increased between 250°D and 350°D during the differentiation of the endosperm and then became heterogeneous with regions of the starchy endosperm appearing unlabeled at maturity (Fig. 2F, G and I).

Using LM19 a signal is detected from 250°D in the outer layers (Fig. 3J). At this stage of wheat grain development, the bright layer corresponding to the testa observed at the mature stage is already labeled. This layer exhibited invaginations with points of intense labeling (Fig. 3J). In the central starchy endosperm a faint LM19 signal was detected from 350°D (Fig. 3K).

**Figure 4:**
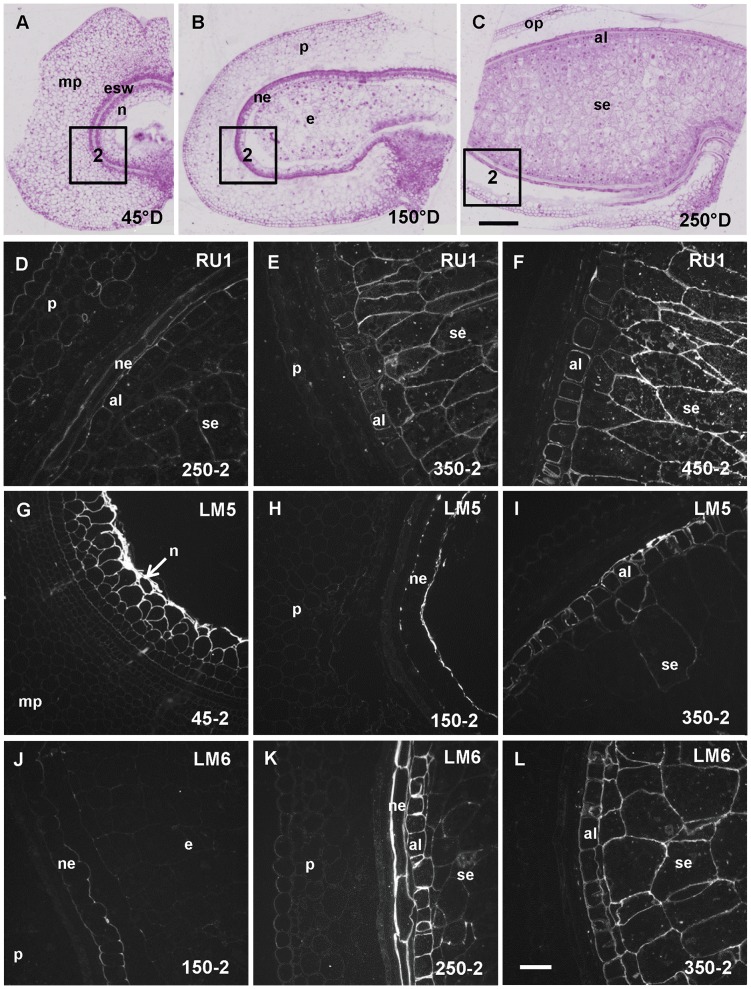
Rhamnogalacturonan I-related epitopes detection in developing wheat grain. A, B, and C. Half wheat grain harvested at 45°D, 150°D and 250°D stained with toluidine blue showing the evolution of the grain tissues and the regions 1 and 2 where the immunofluorescence acquisitions were taken. D, E, F, G, H, I, J, K, L. Immunofluorescence images localizing rhamnogalacturonan I backbone epitope using RUI antibody or galactan and arabinan side chains epitopes using LM5 and LM6 antibodies. The sections were all treated with lichenase and xylanase prior to immunolabeling. D, E, F. RU1 labels weakly most of the cell layers at 250°D, labeling is later confined to the aleurone layer (al) and the starchy endosperm (se). G, H, I. LM5 epitopes are already detected in the nucellus (n) at 45°D. At 150°D, the labeling is localized in the walls of the nucellar epidermis (ne), and later in the development the aleurone layer and starchy endosperm become labeled. J, K, L. LM6 labeling is detected in the nucellar epidermis at 150°D, then in the nucellar epidermis and the the endosperm (aleurone and starchy endosperm) at 250°D and later in the development only in the endosperm. e: endosperm, mp: mesocarp, p: pericarp. Bars represent 250 µm for A, B and C and 50 µm for the remaining images (all at the same scale).

INRA RU1 labeling was detected from 250°D in the pericarp and in the nucellar epidermis (Fig. 4D). A weak signal was detected in the endosperm from 250°D and increased in intensity in the starchy endosperm at 350°D (Fig. 4E). The aleurone layer, which was brightly labeled at 750°D was only weakly labeled at 350°D and 450°D (Fig. 4E and F). LM5 labeled strongly the nucellus at 45°D, then the nucellar epidermis at 150°D (Fig. 4G and H). Labeling in the aleurone layer became obvious from 350°D (Fig. 4I). LM6 labeling was detected from 150°D and only in the nucellar epidermis at this stage (Fig. 4J), while at 250°D LM6 epitopes were detected both in the nucellar epidermis and the endosperm (Fig. 4K). Later in the development the LM6 labeling was restricted to the endosperm (Fig. 4L).

Dramatic changes in the distribution of pectic epitope were observed in the cell walls of the different grain tissues during its development, these changes are summarized in table 1.

**Table 1:** Antibodies used and immunofluorescence results obtained in the wheat grain cell layers during development.

antibody	INRA RU1	LM5	LM6	LM19	LM20
main epitope	unbranched RG	(1–4) β galactan	(1–5) α arabinan	HG	HG
	RGI backbone	RGI side chain	RGI side chain	low-methylated	methylated
**45°D**					
pericarp	−	−	−	−	+
embryo sac wall	−	−	−	−	−
nucellus	−	+	−	−	−
**90°D**					
pericarp	−	−	−	−	+
embryo sac wall	−	−	−	−	+
nucellus	−	+	−	−	−
**150°D**					
pericarp	−	−	−	−	+
nucellus epidermis	−	+	+	−	+
testa	−	−	−	−	−
endosperm	−	−	−	−	−
**250°D**					
outer pericarp	+	−	−	+	+
cross cells	−	−	−	−	+
testa	−	−	−	+	+
nucellus epidermis	+	+	+	−	+
aleurone	+	−	+	−	+
starchy endosperm	+	−	+	−	+
**350°D**					
outer pericarp	−	−	−	+	+
cross cells	−	−	−	−	+
testa	−	−	−	+	−
nucellus epidermis	−	−	−	−	−
aleurone	+	+	+	−	+
starchy endosperm	+	+	+	+	+
**450°D**					
outer pericarp	−	−	−	+	+
cross cells	−	−	−	−	+
testa	−	−	−	+	−
nucellus epidermis	−	−	−	−	−
aleurone	+	+	+	−	−
starchy endosperm	+	+	+	+	+
**750°D (mature)**					
outer pericarp	+	−	−	+	+
cross cells	−	−	−	−	+
testa	−	−	−	+	−
nucellus epidermis	−	−	−	−	−
aleurone	+	+	+	−	−
starchy endosperm	+	−	+	+	+

(+) epitope detected (−) epitope not detected.

### Vesicles filled with HG are detected in the testa at 250°D

**Figure 5:**
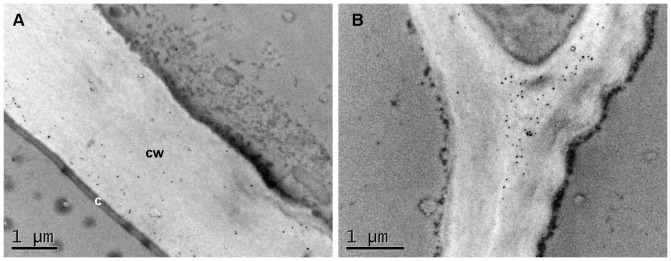
Transmission electron micrograph showing immunogold LM20 detection in the pericarp cell walls. TEM of the outermost cell layer (A) and of a cell junction in the outer pericarp (B) of the wheat grain at 250°D showing labeling in cell wall and in the middle lamella of a cell junction. c: cuticle, cw: cell wall.

To locate more precisely the LM19 and LM20 epitopes in the outer layers and particularly in the testa for which we obtained bright fluorescent signals, transmission electron micrograph (TEM) of grain sections at 250°D were performed. In the pericarp, TEM confirmed the results obtained with immunofluorescence and showed LM20 labeling in the cell wall of the outer pericarp and in middle lamella at cell junctions (Fig. 5).

**Figure 6:**
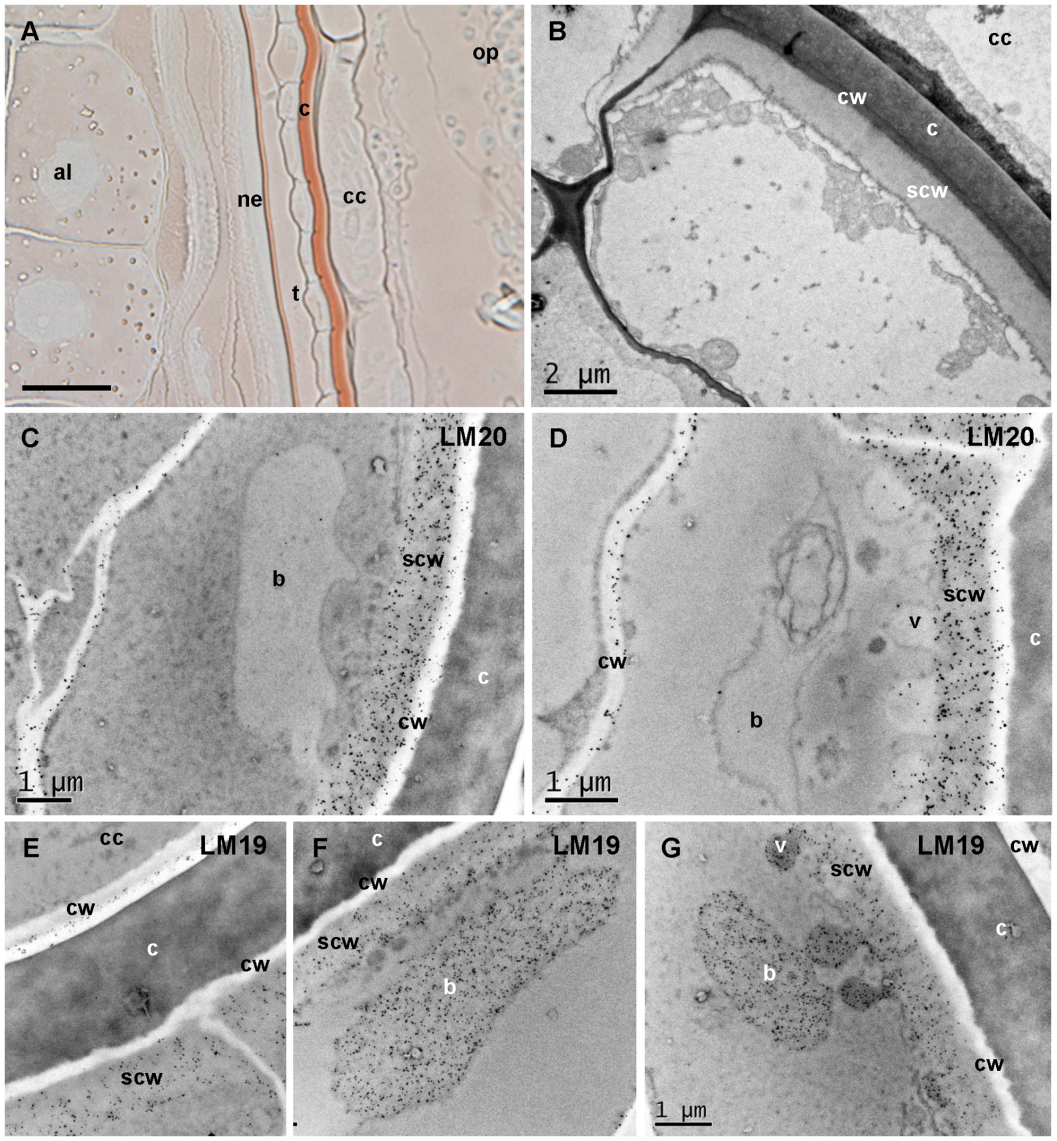
The testa at 250°D secretes homogalacturonans beneath the cuticle with spatial variations in methylesterification. A. Lipophilic Sudan red staining of wheat grain tissues showing the reactive cuticles: a thick cuticle (c) outside the testa (t) and a thinner cuticle outside the nucellar epidermis (ne). The staining is confined to the cuticle and does not react to structures beneath the cell wall of the testa. B. Polysaccharide staining (PATAg) of the outer layer of the testa, the cuticle and the cross cells (cc) showing a thick layer beneath the cell wall (cw) reactive to PATAg. This layer is referred as sub-cell wall layer (scw). C and D. TEM of the outer layer of the testa showing immunogold labeling with LM20 in the sub-cell wall layer, and in the cell wall between the two cell layers of the testa. Large bodies (b) and smaller vesicles (v) not labeled with LM20 are seen. E, F, G. TEM of the outer layer of the testa showing immunogold labeling with LM19 in the sub-cell wall layer, and in the large bodies and vesicles. Bars represent 20 µm in A, 2 µm in B, and 1 µm in C, D, and G (E, F are at the same scale than G).

In the testa, LM20 labeling was mostly localized in the outer cell layer facing the cross cells beneath the cell wall (Fig. 6C and D). Labeling of cell walls was observed. In the same cell layer, LM19 epitopes were not detected in cell walls (Fig. 6E, F and G) but were abundant in large bodies (the largest measured was 13 µm in length in a 20 µm long cell) and smaller vesicles either closed or opened at proximity of the layer labeled with LM20 (Fig. 6F and G). No labeling was observed in these vesicles with LM20 (Fig. 6C and D). Histological staining was used to reveal cell ultrastructures. PATAg staining of polysaccharides revealed a thick layer in the outer cell layer of the testa at 250°D located underneath the outer cell wall (Fig. 6B). This layer corresponds to the structure labeled with LM20. The lipophilic Sudan red stained the seed coat cuticle but not the subcuticle layers (Fig. 6A).

## Discussion

### The wheat grain contains pectin in the endosperm and outer layers

Immunolabeling unambiguously revealed the presence of pectin in the mature wheat grain, both in the outer layers and in the wheat endosperm. Both HG and RGI were detected in mature wheat grain undergoing desiccation and exhibited a specific distribution in the different tissues of the grain. Similar (but not identical) results were obtained with antibodies recognizing RGI backbone and galactan and arabinan confirming that the detected arabinan and galactan are likely to be present as side-chains of RGI. There might be an exception with the nucellus in the early stages only labeled with LM5, which may reveal free galactan. It should be noted that the set of antibodies used in this study does not cover all pectic domains and structures described in plant cell walls.

Until now, no pectin has been reported in wheat grain. In early biochemical studies, the polysaccharide composition of wheat flour, which corresponds approximately to the starchy endosperm, was characterized and did not reveal pectin [3], [5]. We studied the monosaccharide content of grains, flour, as well as isolated outer pericarp and intermediate layers (nucellar epidermis, tube cells, cross cells) from dry grain. Traces of pectic components were detected. A prior report of traces of galacturonic acid in wheat bran, which corresponds to the pericarp and the aleurone layer, was published by Beaugrand et al. [29]. All these results are in favor of the presence of pectin in the wheat grain. We detected pectin in the wheat grain but obviously the overall amount must be small.

Immnunolabeling is a very sensitive technique, much more sensitive than the colorimetric/biochemical detection assays used in prior attempts. Immunolabeling enables the detection of low abundant molecules and the study of the distribution of this molecule in different tissues and cell-types. Accessibility of the epitope is a major issue for immunolabeling. Cell wall epitopes may be masked by abundant polymers. Several studies have already reported that cell wall epitope masking may be overcome by enzymatic pretreatment [27], [28]. In our present work, we conducted a preliminary degradation of arabinoxylans and mixed-linked beta-glucans that allowed to reveal pectic epitopes particularly in the endosperm. This result may explain that the presence of pectin domains within the wheat endosperm has been underestimated until now [7]. Epitope masking may not be the only cause. Complex linkages between cell wall components may hinder epitope detection and may contribute to signal heterogeneity. Tissue preparation and embedding medium may also influence immunodetection.

### Pectic domains are differentially and specifically distributed and methylated depending on cell types and development stages

Interestingly, signals for pectin domains were not always superimposed revealing cell layers where RGI is more abundant such as aleurone cells and others such as the outer layers where HG is prevalent. This fact challenges the model describing HG as a side chain of RGI [30].

LM19 and LM20 antibodies were used to determine whether the wheat grain HG glycans were methylated. Indeed LM19 binds strongly to unesterified HG whereas LM20 binds to esterified HG and not to unesterified HG. Our results suggest that methylated HG are present in the grain (pericarp) from the early stages of development since we detected them at 45°D (2 DPA). At this stage no signal was revealed for unmethylated HG. Methylesterification of HG occurs at the C-6 position of galacturonic acid residues and affects the charge of the polymer and the physical properties of cell walls (e.g. elasticity, extensibility) [16]. The current model proposes that HG is synthesized and delivered to the cell wall in a highly methylated form to hinder chain interactions through calcium bridges [31], [32]. These highly methylated HG would promote elasticity of cell wall in expanding cells such as wheat expanding pericarp and developing endosperm. In differentiated tissues where expansion stopped (in late development stages) the occurrence of unmethylated HG produced by pectin methylesterases would decrease the wall extensibility. In these tissues, our results fit the proposed model.

### The wheat testa secretes a layer of HG beneath the cuticle

At 250°D in the outer cell layer of the seed coat, methylated and low or unmethylated HG were detected in a thick and dense layer beneath the cuticle. In the subsequent stages however, only epitopes corresponding to low or unmethylated HG were detected. At 250°D, massive bodies filled with low or unmethylated HG and smaller vesicles delivering HG to the subcuticle layer were observed. From our present data, we cannot conclude whether these bodies are synthesizing low or unmethylated HG -which would contrast with the current model of HG synthesis -or whether they are producing methylated HG together with pectin methyl esterases that concomitantly remove methyl esters. The thick and dense layer of matter underneath the cuticle and the testa cell wall are reactive to PATAg staining revealing polysaccharides but not to the lipophilic Sudan red, which suggests that they do not contain cutin or suberin.

The testa derives from the wall of the embryo sac, precisely from the inner integument [33]. During grain growth, the other layers of integuments degenerate, not the testa. The testa of many species synthesizes pectin. Arabidopsis seed coat synthesizes and secretes massively pectin in the form of a mucilage which consists mostly in RGI with only a small fraction of HG and other polysaccharides [34]. The cell wall covering the columella where the mucilage is stored contains methylated HG and a mutant with decreased degree of methylated HG showed defect in the mechanical properties of the cell wall and therefore in the wall rupture required to release the seed mucilage [35].

In contrast with Arabidopsis seed coat, wheat seed coat is not exposed directly to the environment as it is surrounded by the pericarp. Nevertheless, the wheat testa undergoes major changes during wheat grain development and especially a thick cuticle is deposited on its outer surface [33]. Two other thinner cuticles are formed in the outermost layer of the pericarp and outside the nucellar epidermis [33] but the cell layers underneath did not exhibit such a massive pectin deposition at the investigated stages. The cuticle is a complex structure found in many plant organs (e.g. leaf, fruit and seed) containing waxes and cutin, a polyester of hydroxy and fatty acids. Plant cuticles are associated with polysaccharides including pectin in Arabidopsis seed, in developing cotton fibers and leaf trichomes [36], and in tomato fruit [37]. Our findings highlights that even in plants with overall only traces of pectin, specific cell layers can massively synthesize pectin presumably to fulfill a function that has yet to be established.

### Correlation between biosynthetic enzyme families identified in the grain and polysaccharide composition

The discovery of pectin in wheat grain explains the presence of transcripts and proteins presumably involved in the synthesis of pectin in the wheat grain endosperm [7], [14]. It has been postulated that the number of activities required to make complex pectin may reach 67 [15]. So far the GT families implicated in pectin synthesis are GT8, GT47, GT77 and GT92. The Arabidopsis GT8 GALACTURONOSYLTRANSFERASE 1 (GAUT1) was shown to be an α-galacturonosyltransferase involved in the synthesis of the HG backbone [38]. Among the 41 members of the GT8 family in Arabidopsis only the GAUT clade is thought to be involved in the pectin synthesis [39]. Pellny et al [7] detected by RNAseq transcripts for 16 GT8 genes in the wheat endosperm. By localizing the rice orthologs of their wheat sequences in the phylogenetic analysis done by Yin et al. [39] we determined that among these 16 GT8 genes 12 are GAUT genes (Table 2). GT47 is also a family of GT with many members [40] and several cell wall related activities are associated with the different clades of the family. Two clades are implicated in the synthesis of pectin. A xylogalacturonan xylosyltransferase activity was proposed for the GT47 XGD1 (XYLOGALACTURONAN DEFICIENT) [41] and several GT47 would be arabinan arabinosyltransferase, (ARAD ARABINAN DEFICIENT [42]–[43]. Both our proteomic survey and Pellny's transcriptomic survey of the wheat endosperm revealed members of the GT47 clade implicated in pectic arabinan synthesis. By proteomics we also identified wheat proteins with similarity to GT77, GT92 and Carbohydrate esterase family 13 respectively implicated in the synthesis of RGII and pectic galactans and in the desacetylation of pectin which would imply that wheat pectin is acetylated as reported for many other species [14], [44], [45], [19].

**Table 2:** GT8 genes detected in the developing wheat endosperm.

Pellny et al 2012	Rice ortholog	clade
**TaGT8_1**	LOC_Os09g36190	GAUT
**TaGT8_2**	LOC_Os07g48370	GAUT
**TaGT8_3**	LOC_Os06g49810	GAUT
**TaGT8_4**	LOC_Os12g38930	GAUT
**TaGT8_5**	LOC_Os02g41520	PGSIP
**TaGT8_6**	LOC_Os03g30000	GAUT
**TaGT8_8**	LOC_Os08g23780	GAUT
**TaGT8_7**	LOC_Os06g12280	GAUT
**TaGT8_9**	LOC_Os10g40640	GAUT
**TaGT8_10**	LOC_Os04g43700	PGSIP
**TaGT8_11**	LOC_Os04g54360	GAUT
**TaGT8_12**	LOC_Os03g11330	GAUT
**TaGT8_13**	LOC_Os09g30280	GAUT
**TaGT8_14**	LOC_Os02g51130	GAUT
**TaGT8_16**	LOC_Os02g50600	GATL
**TaGT8_17**	LOC_Os09g36180	GAUT

GAUT (Galacturonosyltransferase), GATL (GAUT-like), PGSIP (plant glycogenin-like starch initiation proteins).

The pectin present in the wheat endosperm may thus contain the different known pectic domains. The current work shows that it may be possible to anticipate the cell wall components of a target tissue/species based on the GT families identified in the sample.

### Conclusions

Pectic polysaccharides are components of the endosperm cell walls in many species even in Arabidopsis where the endosperm is restricted to a single layer surrounding the embryo [46]. In investigated grass grains and in particular in the endosperm, pectin seems to be present [9]–[10]. Ignored in many cases, pectin was not reported in barley endosperm [8] and previously not detected in wheat endosperm. The significance of pectin occurrence in grass grain is difficult to apprehend and more experiments are required (e.g. producing grain without pectin) to assert the functions of pectin in the grain. It becomes obvious that the cell walls in the wheat grain are not merely an association between arabinoxylans and mixed-linked glucans, since these walls contain as well mannans, cellulose, xyloglucans, pectin and possibly arabinogalactan proteins although the location of wheat arabinogalactan proteins is still under debate [6]. The roles of the minor polymers in the grain cell wall are unknown but recent discoveries in other species foresee important functions in cell wall architecture and properties and in external signal sensing and transduction [47], [48].

## Supporting Information

Figure S1 **Effect of pectin lyase on the structure of testa revealed by UV autofluorescence (750°D.**
(PPTX)

Figure S2 **Xylanase and lichenase treatments drastically decreased xylan and mixed-linked glucan labeling.**
(PPTX)

Figure S3 **Sodium carbonate treatment increased LM19 labeling.**
(PPTX)

Figure S4 **Monosaccharide content in wheat dry grain, flour, isolated outer pericarp and intermediate layers.**
(PPTX)

Figure S5 **Immunolabelings conducted without xylanase and lichenase treatments.**
(PPTX)

Figure S6 **DIC images corresponding to the immunofluorescence images of** **Figures 1** **,** **2** **,** **3** **and** **4** **.**
(PPTX)
